# The importance of superficial basal cell carcinoma in a retrospective study of 139 patients who underwent Mohs micrographic surgery in a Brazilian university hospital

**DOI:** 10.6061/clinics/2015(11)01

**Published:** 2015-11

**Authors:** Luciana Takata Pontes, Rafael Fantelli Stelini, Maria Leticia Cintra, Renata Ferreira Magalhães, Paulo Eduardo N.F. Velho, Aparecida Machado Moraes

**Affiliations:** I Hospital das Clínicas da Universidade Estadual de Campinas, Clínica Médica, Divisão Dermatologia; II Hospital das Clínicas da Universidade Estadual de Campinas, Patologia, São Paulo/SP, Brazil

**Keywords:** Mohs Surgery, Carcinoma, Basal Cell, Skin neoplasms

## Abstract

**OBJECTIVE:**

Mohs micrographic surgery is a specialized surgical procedure used to treat skin cancer. The purpose of this study was to better understand the profile of the patients who underwent the procedure and to determine how histology might be related to complications and the number of stages required for complete removal.

**METHODS:**

The records of patients who underwent Mohs micrographic surgery from October 2008 to November 2013 at the Dermatology Division of the Hospital of the Campinas University were assessed. The variables included were gender, age, anatomical location, histology, number of stages required and complications.

**RESULTS:**

Contingency tables were used to compare the number of stages with the histological diagnosis. The analysis showed that patients with superficial basal cell carcinoma were 9.03 times more likely to require more than one stage. A comparison between complications and histological diagnosis showed that patients with superficial basal cell carcinoma were 6.5 times more likely to experience complications.

**CONCLUSION:**

Although superficial basal cell carcinoma is typically thought to represent a less-aggressive variant of these tumors, its propensity for demonstrating “skip areas” and clinically indistinct borders make it a challenge to treat. Its particular nature may result in the higher number of surgery stages required, which may, as a consequence, result in more complications, including recurrence. Recurrence likely occurs due to the inadequate excision of the tumors despite their clear margins. Further research on this subtype of basal cell carcinoma is needed to optimize treatments and decrease morbidity.

## INTRODUCTION

Mohs micrographic surgery (MMS) is a specialized surgical procedure used to treat skin cancer that is commonly performed on patients who present with a large tumor (>2 cm), a high-risk histological type tumor (for example, morpheaform, infiltrative or micronodular basal cell carcinoma (BCC), poorly differentiated squamous cell carcinoma (SCC), or a tumor with perineural invasion), recurrent or incompletely excised tumors, tumors located in the H zone of the face and lesions with indistinct clinical borders 1,2.

This technique histologically maps the margins of the tumor infiltration into the tissue while sparing healthy tissue, which allows greater tissue conservation and margin control compared with other surgical procedures 2. After mapping the excised tissue and the surgical defect, the specimens are frozen and stained to make histologic slides. If neoplastic cells are detected on the slides, more tissue is excised at the exact location of the remaining tumor tissue. The procedure is complete when no evidence of neoplastic cells is detected in the frozen sections.

One of the objective measures of an MMS case is the number of stages required for tumor clearance 3. This number is believed to be related to numerous factors, including the anatomical site of the tumor; for example, those located in cosmetically sensitive areas, such as the nose tip and eyelid, can potentially require more stages. Other factors include tumor histology, as in morpheaform BCC, which displays a wider-than-visualized lateral spread in the dermis 4-6. Tumor size alone is not believed to predispose the patient to requiring more stages and well-demarcated tumors can be removed in only one or a few stages 4.

As with any surgical procedure, there is a risk of post-operative complications, most of which involve bleeding 1. Other complications that have been described include infection, flap or graft loss, unfavorable contours, scars and delayed healing 5. In this study, we considered recurrence to be a late complication.

The purpose of this study was to better understand MMS patient profiles and to determine how the histology of tumors might be related to the number of MMS stages required and the complications of the procedure.

## MATERIALS AND METHODS

The records of all of the patients who underwent MMS from October 2008 to November 2013 at the Dermatology Division of the Clinical Hospital at Campinas University (HC-UNICAMP) were assessed.

If not all of the information necessary for the study was included in a patient's records, the patient was excluded.

The variables included were gender, age, anatomical location, histology of the tumor, number of stages and complications.

Exploratory data analysis (EDA) was performed using a frequency calculation. Logistic regression models were applied to evaluate the risk factor for each response variable of interest. For multiple factor analyses, a *stepwise* method was chosen to select the variables. A 95% confidence level was used and the statistical analyses were performed using the Statistical Analysis System for Windows software, version 9.4 (SAS 9.4).

## RESULTS

A total of 139 patients comprising 55 (39.57%) males and 84 (60.43%) females were included. Of these patients, 14 (10.07%) were 40 years or younger, 21 (15.11%) were 41 to 50 years old, 26 (18.71%) were 51 to 60 years old, 34 (24.46%) were 61 to 70 years old and 44 (31.65%) were 71 or older (Table 1).

Table 2 presents the descriptive statistics for the anatomical location of the tumor. The nose was the most frequent location, comprising 61.9% of the total patients, followed by the periocular area, the nasolabial fold, the perilabial area, the temporal/frontal area or the glabella, malar and ear.

Each tumor was histologically classified according to a prior biopsy conducted previous to the MMS and then classified as nodular BCC, superficial BCC, infiltrative-growth BCC, morpheaform BCC, micronodular BCC, non-specified BCC, SCC, trichoblastoma, dermatofibrosarcoma protuberans (DFSP), fibrous histiocytoma and primary cutaneous adenocarcinoma. Some lesions contained tissue of more than one histological type in the same sample and this was taken into account in our study (Table 3).

The patients underwent one to four surgery stages, with four being the maximum number of stages required in all of the included cases. Most of the patients (52.52%) underwent only one MMS stage (Table 1).

The classification of complications was determined according to postoperative results. They were divided into no complication, bleeding, ectropion, microstomy, flap or graft necrosis and recurrence (Table 1). We considered recurrence a late complication to facilitate the study of the variables.

There were four patients who experienced recurrence: two had a superficial BCC (50%), one had a nodular BCC (25%) and one had a morpheaform and infiltrative BCC (25%).

Contingency tables were used to compare the number of MMS stages with the histological diagnosis. Table 4 presents the simple and multiple logistic rank regressions used to correlate the tumor histology with the number of stages (*p*-value<0.05). The individual analysis showed that patients with a superficial BCC were 9.03 times more likely to require more stages of surgery. When the risk factors were studied in combination (multiple analysis), it was confirmed that the patients with an increased risk of a higher number of MMS stages were those who were diagnosed with superficial BCC. The histological diagnoses that do not appear in Table 4 were excluded due to the low number of cases.

Contingency tables were used to compare complications with the histological diagnosis and age. Table 5 represents the simple and multiple binary logistic regressions used to evaluate the risk factors associated with complications (*p*-value<0.05). In the individual analysis, it was observed that patients with superficial BCC were 6.5 times more likely to experience complications.

Multiple analyses confirmed that superficial BCC was a risk factor for complications and that there was no correlation between complications and other histological diagnoses or age. The histological diagnoses that do not appear in Table 5 were not used due to the low number of cases or because the patients did not present any post-operative complications.

## DISCUSSION

Because MMS surgery results in a high cure rate and maximal preservation of the normal tissue, it is a valuable treatment for many modalities of skin cancer. The cure rate for patients who undergo MMS has been observed to be 99% for primary BCC and 93% to 98% over a 3- to 5-year time frame for recurrent BCC in several retrospective studies 1,6,7.

**Figure 1:**
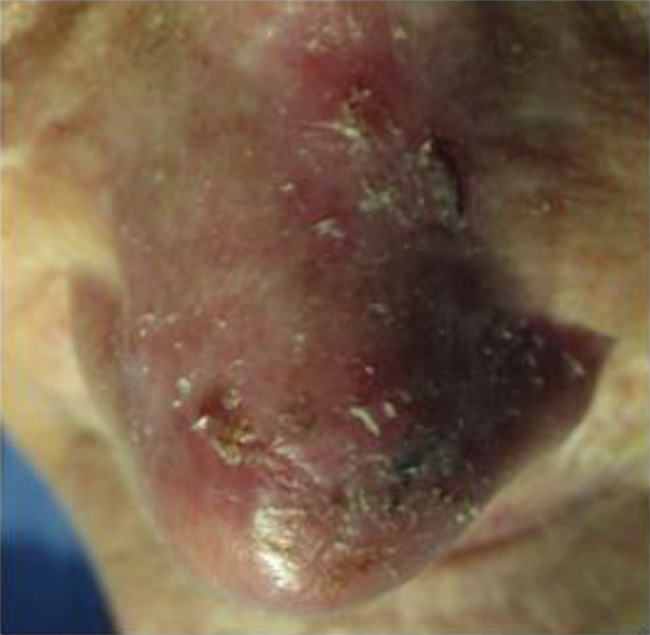
Superficial basal cell carcinoma of the nose.

Superficial BCC is often considered to be a non-aggressive form of BCC and it has been hypothesized that this type of BCC has an increased likelihood of developing in the trunk, as it often develops after intense but intermittent sun exposure 8. Clinically, it tends to present as thin, pink, scaly plaques that often have indistinct borders (Figure 1).

**Figure 2:**
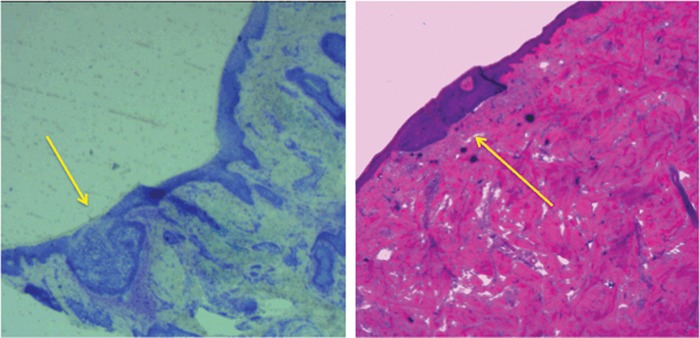
Histological slides: Superficial basal cell carcinoma (yellow arrow). Left: toluidine blue stain. Right: hematoxylin and eosin stain.

Histologically, it is unique because it consists of small nest of basaloid cells connected to the epidermis with minimal dermal involvement (Figure 2). The nests are intermixed with normal, non-involved epidermis in a multifocal pattern. These “skip areas” are probably what make this type of skin cancer so difficult to treat 8.

The aggressive subtypes of BCC (infiltrative, morpheaform, micronodular and metatypical) are known to require more MMS stages to achieve tumor-free margins 3,9. Batra et al. published a retrospective analysis of 1095 MMS cases that displayed high-risk factors that were associated with extensive tumor spread, which was defined as a requirement of at least 3 MMS stages. It was determined that the predictive characteristics included any BCC on the nose, morpheaform BCC on the cheek, neck tumors and recurrent BCC in men, location on the eyelid, temple, or ear helix and a size>10 mm 10.

In our study, we found the superficial BCC tumor type requires a higher number of stages for removal. We believe that the main reason for this finding is the difficulty of clinically delimiting the tumors in these cases, which probably reflects the fact that the tumors tend to spread widely and to include “skip areas”, where the tumor is present but is sometimes not as clinically visible as a typical lesion (thin, pink, and scaly plaques). This fact probably results in the surgeon removing a narrow initial margin in the first stage of the MMS.

Sexton et al. described a series of 1039 consecutive neoplasms in patients who underwent conventional surgery and found that nodular and superficial basal cell carcinomas were removed by simple surgical excisions in a high percentage of these cases (93.6% and 96.4%) 11. This result is probably because the patients underwent conventional surgery and not MMS. During conventional surgery, the surgeon tends to remove a larger margin (more than 4 mm), mainly in areas where there is no need to save tissue, such as the trunk, where superficial BCC is more common 8. In contrast, when performing MMS, the surgeon removes a 1 to 2 mm margin from the clinical lesion, making tumors with indistinct clinical borders a challenge to excise in one stage.

The correlation between superficial BCC and complications was probably due to the higher number of stages required for removal, which increases the length of the surgery, resulting in a higher probability of bleeding and therefore an increased likehood of necrosis.

In this study, recurrence was considered a late complication. Of the four cases of recurrence, two were superficial BCC. Recurrence probably occurs due to the inadequate excision of these tumors because the “skip areas” can create the false impression of a free margin.

Some authors defend the use of topical 5% imiquimod or photodynamic therapy (PDT) as an alternative treatment for superficial BCC, especially for large superficial types and multiple lesions and for patients who have contraindications to surgical intervention 12-14. Although these less-aggressive treatment options are available for patients with low-risk BCC, surgery is still regarded as the gold standard 15-17.

Imiquimod 5% cream is a topical immune response modifier that was approved by the US FDA in 1997 for the treatment of genital and perianal warts 18. Its mechanism of action is thought to involve the activation of macrophages and other cells via binding to cell-surface receptors such as the Toll-like receptor 7 (1). This stimulation of the innate and cell-mediated immune responses leads to the generation of antitumor and antivirus activity and it has been studied as a treatment for superficial BCC, pigmented BCC and other cutaneous neoplasms 18. Schulze et al. conducted a randomized controlled trial of 166 patients that compared the effects of 6 weeks of daily imiquimod administration to those of a placebo. Histological tests at 12 weeks post-treatment demonstrated that imiquimod displayed improved efficacy over the placebo for treating superficial BCC (the histologic clearance rate was 80% *vs*. 6%) 19.However, Bath-Hextall et al. showed that the application of topical imiquimod was inferior to surgery and presented no clear cosmetic or cost benefits 15.

PDT is based on light activation of a photosensitizer localized on the tumor tissue to generate highly reactive oxygen intermediates that irreversibly oxidize essential cellular components. This process causes tissue damage and necrosis and induces apoptosis 20. Three randomized controlled studies with long-term follow ups have compared the various effects of PDT protocols to those of simple surgical excision protocols for treating superficial BCC and primary nodular BCC. All of these studies have demonstrated statistically superior clearance rates for surgical excision over PDT 21-23.

As an option for obtaining better results and decreasing morbidity, some authors have considered the possibility of combined treatments that use topical chemotherapy with MMS 24,25. Because this type of tumor can recur despite a surgical excision with negative margins (because of its tendency to produce a mosaic pattern with tumor-free regions), we believe that association therapy is a good option for treating superficial BCC. The topical treatment could be used before MMS to reduce the size of the defect requiring removal and the number of stages required during surgery and it could be used after surgery to clear residual tumor nests. Torres et al. reported on a randomized, double blind, vehicle-controlled study of imiquimod administered 5 days per week for 2 to 6 weeks before MMS excision to remove superficial BCC and nodular BCC. They showed that imiquimod reduced the size of the target tumor and thereby resulted in a smaller defect to be excised during MMS in subjects in the 4- and 6-week dosing regimens compared to the vehicle 26.

In conclusion, superficial BCC is unique, given its propensity for “skip areas”, recurrence and larger defects than would be clinically expected. Further research is needed to optimize treatment for these tumors and to decrease morbidity in these patients.

## Figures and Tables

**Table 1:** Descriptive statistics.

Variable	N	%
Gender		
female	84	60.43%
male	55	39.57%
Age		
≤40	14	10.07
41-50	21	15.11%
51-60	26	18.71%
61-70	34	24.46%
≥71	44	31.65%
Stages		
1	73	52.52%
2	50	35.97%
3	13	9.35%
4	3	2.16%
Complications		
none	130	93.5%
recurrence	4	2.87%
bleeding	1	0.72%
ectropion	1	0.72%
microstomy	1	0.72%
flap or graft necrosis	2	1.44%

**Table 2:** Anatomic location descriptive statistics.

Anatomical Location	Frequency	% of patients (n=139)	% of the total anatomical locations (n=145)
Nose	86	61.9	42.7
Periocular	24	17.3	11.9
Nasolabial fold	9	6.5	4.5
Perilabial	8	5.8	4.0
Temporal, frontal or glabella	6	4.3	3.0
Malar	5	3.6	2.5
Other	4	2.9	2.0
Ear	3	2.2	1.5

**Table 3:** Descriptive statistics by histological diagnosis.

Histological diagnosis	Frequency	% of patients (n=139)	% of the histological diagnoses (n=199)
Nodular BCC	66	47.5	23.9
Infiltrative-growth BCC	47	33.8	17.0
Morpheaform BCC	39	28.1	14.1
Superficial BCC	16	11.5	5.8
Non specified BCC	15	10.8	5.4
Micronodular BCC	7	5.0	2.5
SCC	4	2.9	1.4
DFSP	2	1.4	0.7
Trichoblastoma	1	0.7	0.4
Fibrous histiocytoma	1	0.7	0.4
Primary cutaneous adenocarcinoma	1	0.7	0.4

BCC: basal cell carcinoma; SCC: squamous cell carcinoma; DFSP: dermato-fibrosarcoma protuberans

**Table 4:** The logistic regression results used to evaluate the association between the histologic diagnosis and number of stages.

Simple analysis					
Factor	Effect *vs.* Ref	Odds ratio	95% CI (odds ratio)		*p-*value
Nodular BCC	Y *vs*. N	1.784	0.930	3.422	0.0814
Superficial BCC	Y *vs*. N	9.03	3.104	26.244	<0.0001
Infiltrative BCC	Y *vs*. N	1.299	0.658	2.566	0.4508
Morpheaform BCC	Y *vs*. N	1.497	0.732	3.062	0.2692
Micronodular BCC	Y *vs*. N	0.914	0.208	4.020	0.9052
Non-specified BCC	Y *vs*. N	0.382	0.117	1.243	0.1099

Multiple analysis					
Factor	Effect *vs.* Ref	Odds ratio	95% CI (odds ratio)		*p-*value
Superficial BCC	Y *vs*. N	9.03	3.104	26.244	<.0001

The histologic diagnoses that do not appear in the table were excluded due to the low number of cases.

**Table 5:** The results of the binary logistic regression used to evaluate factors associated with complications.

Simple analysis						
Factor	Effect *vs*. Ref	Odds ratio	IC 95% (Odds ratio)			*p*-value
Nodular BCC	Y *vs*. N	1.115	0.308	4.037	0.8686
Superficial BCC	Y *vs*. N	6.500	1.607	26.298	0.0087
Infiltrative BCC	Y *vs*. N	1.333	0.357	4.977	0.6685
Morpheaform BCC	Y *vs*. N	1.790	0.477	6.725	0.3883
Micronodular BCC	Y *vs*. N	–	–	–	–
Non-specified BCC	Y *vs*. N	–	–	–	–
Age	≥71 *vs*. ≤70	2.308	0.632	8.428	0.2057

Multiple analysis						
Factor	Effect *vs*. Ref	Odds ratio	95% CI (Odds ratio)		*p*-value
Superficial BCC	Y *vs*. N	6.500	1.607	26.298	0.0087

The histologic diagnoses that do not appear in the table were excluded due to the low number of cases or because the patients did not present any post-operative complications.
